# Ventral-inlay buccal mucosal graft urethroplasty in a 44-year old female patient with recurrent urethral stricture

**DOI:** 10.1093/jscr/rjad025

**Published:** 2023-02-06

**Authors:** Orlando Burkhardt, Hans-Peter Schmid, Daniel Engeler, Valentin Zumstein

**Affiliations:** Department of Urology, School of Medicine, University of St. Gallen, St. Gallen, Switzerland; Department of Urology, School of Medicine, University of St. Gallen, St. Gallen, Switzerland; Department of Urology, School of Medicine, University of St. Gallen, St. Gallen, Switzerland; Department of Urology, School of Medicine, University of St. Gallen, St. Gallen, Switzerland

## Abstract

Female urethral stricture is a rare manifestation of bladder outlet obstruction in women. According to the current guidelines of the European Association of Urology, urethral dilatation should be offered as first line treatment. Intermittent self-dilatation (ISD) in case of recurrence is recommended. However, if patients wish definitive surgical treatment or are not able to perform ISD, urethroplasty can be considered. So far, there are little data available on urethroplasty in female patients. We present a case of a 44-year old female patient with a postoperative urethral stricture who underwent ventral-inlay buccal mucosal graft urethroplasty due to inability to perform ISD.

## INTRODUCTION

Female urethral strictures account for ~4–13% of women with bladder outlet obstruction and therefore is thought to be a rare disease [1]. Though, since strictures present with frequency, urgency, poor flow, incomplete bladder emptying and consecutive urinary tract infections, quality of life can be severely impaired. According to the guidelines of the European Association of Urology (EAU) dilatation of the urethra up to 30–41 Fr with subsequent intermittent self-dilatation (ISD) or planned repeated dilatation in case of recurrence is the recommended first line treatment strategy [1–4]. However, in patients with recurrent strictures who wish definitive treatment and/or are unable to continue ISD due to physical inability or pain, urethroplasty can be performed. A variety of techniques, primarily using grafts, have been proposed [2, 5]. Currently, there is no recommendation on which technique should be performed. In fact the technique of urethroplasty should be chosen according to surgeons expertise, availability and quality of the graft material. Since female urethral strictures are rare and the vast majority of symptomatic patients is treated by dilatation without any further surgical interventions, we present a case in which we performed a ventral-inlay buccal mucosal graft urethroplasty.

## CASE REPORT

A 44-year old female patient presented with frequency, urgency and severe painful micturition 1 year after excision of a urethral caruncle. Clinical examinations revealed a reduced urinary flow rate (Qmax 7.6 ml/s), relevant post void residual volume of 110 ml and an International Consultation on Incontinence Questionnaire Female Lower Urinary Tract Symptoms Score (ICIQ-FLUTS Score) of 22 points (filling symptoms 10 points, voiding symptoms 12 points and incontinence symptoms 0 points). Urethrocystoscopy showed a pre sphincteric stricture that could not be passed with the 17 Fr flexible cystoscope. Therefore, dilatation of the urethra up to 30 Fr was performed. After the procedure, urinary flow rate was sufficient and frequency/urgency dissolved completely for a few days. Unfortunately, the stricture recurred 2 weeks later and the patient complained about the same initial symptoms. ISD was no treatment option in this patient due to severe pain during dilatation. We therefore discussed a ventral-inlay buccal mucosal graft urethroplasty for definitive treatment.

**Figure 1:**
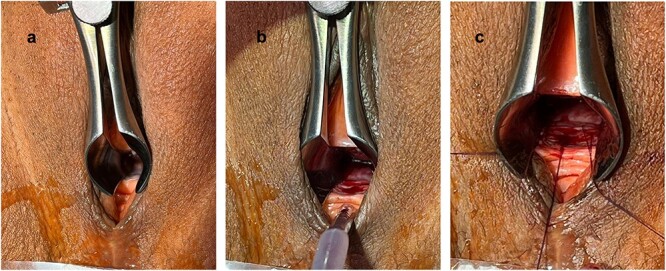
Exposure of the urethral stricture using a nasal speculum (**a**); after urethrotomy at 6 o’clock (**b**); prepared 5-0 PDS sutures at 5, 6 and 7 o’clock (**c**).

**Figure 2:**
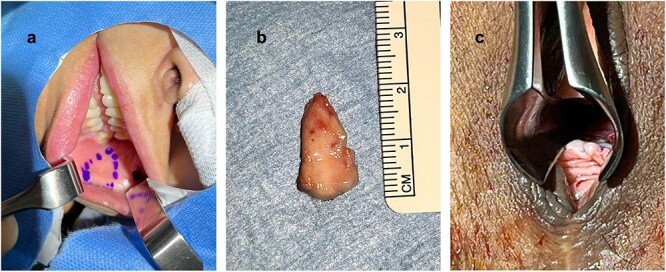
Planning of buccal mucosal graft harvesting (**a**); harvested buccal mucosal graft (**b**); after complete fixation of the graft (**c**).

The patient was placed in a lithotomy position and a nasal speculum was used to expose the extend of the urethral stricture (Fig. 1a). A ventral urethrotomy at 6 o’clock was performed using a 15-blade scalpel transecting the urethral mucosa and the underlying fibrotic tissue creating an ~1.5 cm long triangular defect (Fig. 1b). Three 5-0 polydioxanone suture (PDS) simple interrupted sutures were prepared at 5, 6 and 7 o’clock (Fig. 1c). The normal 5-0 PDS needle was bend into a J-shape to facilitate taking stitches. An ~2 cm × 1.5 cm buccal mucosal graft was harvested and prepared for ventral-inlay fixation (Fig. 2a and b). The graft was placed and fixed to the urethra using the three prepared sutures. Additional lateral stitches were taken for definitive fixation (Fig. 2c). A 12-Fr silicon Foley’s catheter was placed to drain the bladder. The catheter remained in place until Day 10 after the procedure.

After catheter removal on Day 10, the patient showed a maximum urinary flow rate of Qmax 28 ml/s. Post void residual volume decreased from 110 ml preoperatively to 50 ml. At 6 weeks Qmax remained stable and postvoid residual volume decreased to 15 ml. ICIQ-FLUTS score decreased from 22 to 7 points (filling symptoms 1 point, voiding symptoms 0 points, incontinence symptoms 6 points). Unfortunately, the patient reported a slight postoperative urge incontinence with the need of 1 pad per 24 h. The patient had a history of overactive bladder and received intravesical Botox injection 2 years before the buccal mucosal graft urethroplasty. Therefore, we planned another injection 2–3 months postoperatively. In addition, pelvic floor physiotherapy was instructed. However, there were no signs of postoperative stress urinary incontinence. Thus, we consider the postoperative incontinence rather associated with the overactive bladder than as a postoperative symptom after buccal mucosal graft urethroplasty.

## DISCUSSION

A variety of techniques of surgical management of female urethral strictures have been proposed. However, there is no recommendation on which technique should be performed in each case [1]. Nayak *et al*. [5] presented a small case series of 12 female patients with urethral strictures undergoing a ventral-inlay buccal mucosal graft urethroplasty. They reported a success rate of the procedure of 92% within a follow-up period up to 28 months and discussed several potential advantages of the ventral-inlay approach. Since vaginal and urethral dissection and manipulation are not necessary in this technique, postoperative pain and the risk of urethrovaginal fistula is reduced compared to other techniques. Furthermore, compared to the dorsal approach, transection of the pubourethral ligament can be avoided and therefore lower rates of postoperative stress urinary incontinence can be expected. However, long-term follow data are still missing. In our patient suffering primarily from painful micturition, urgency and frequency, we aimed to choose a technique with as little manipulation as possible and decided to perform a ventral-inlay buccal mucosal graft urethroplasty. The procedure in our patient showed excellent functional outcome with a sufficient maximum urinary flow rate, absence of pain and no urinary incontinence 6 weeks postoperatively.

Ventral-inlay buccal mucosal graft urethroplasty seems to be a safe and feasible alternative to the recommended first line treatment with dilatation in female patients with recurrent urethral strictures and/or inability to perform ISD.

## CONFLICT OF INTEREST STATEMENT

None declared.

## FUNDING

None.

## DATA AVAILABILITY

The data underlying this article are available in the article.
